# Dietary patterns in internal migrants in a continental country: A population-based study

**DOI:** 10.1371/journal.pone.0185882

**Published:** 2017-10-16

**Authors:** Antonio Augusto Ferreira Carioca, Bartira Gorgulho, Juliana Araujo Teixeira, Regina Mara Fisberg, Dirce Maria Marchioni

**Affiliations:** Department of Nutrition, School of Public Health, University of Sao Paulo, Sao Paulo, Brazil; Leibniz-Institut fur Pflanzengenetik und Kulturpflanzenforschung Gatersleben, GERMANY

## Abstract

**Objective:**

The objective of this study was to assess the differences and similarities in dietary patterns among migrants and natives.

**Methods:**

A population-based, cross-sectional study was conducted in the city of São Paulo. The study population included internal migrants, defined as individuals born outside São Paulo city who had lived in the city for ten years or longer. The final population (*n* = 999) was divided into three groups: natives of São Paulo (*n* = 354), migrants from the Southeast (*n* = 349) and migrants from the Northeast (*n* = 296). Factor and principal component analysis was employed to derive dietary patterns. The standardized scores were compared among groups using linear regression.

**Results:**

Differences in income per capita, years of education, self-reported race, smoking habits, alcohol consumption, nutritional status and prevalence of hypertension were found for place of birth. Three dietary patterns were identified: prudent (salad dressings, vegetables, natural flavorings, fruits, whole-grain bread, white cheeses and juices), traditional (rice, beans, bread/toast/crackers, butter/margarine, whole milk, coffee/teas, sugar), and modern (sodas, pastries/sandwiches/pizzas, yellow cheeses, pastas, sauces, alcoholic beverages, sweets, processed meats). Compared to natives, migrants from the Southeast had an inversely proportional adherence to the modern pattern whereas migrants from the Northeast had an inverse association with the prudent and modern patterns and a positive association with the traditional pattern.

**Conclusions:**

São Paulo natives and internal migrants from other regions of Brazil exhibited different dietary patterns. The results presented here add perspectives to be considered in the study of non-communicable diseases and its different incidences among migrants and natives.

## Introduction

The study of migrant populations has been pivotal to broadening understanding on the etiology of chronic diseases [1–5]. Exposure to different environmental, cultural and socioeconomic settings can promote differences in individuals´ dietary patterns and metabolic profiles. In Brazil, a greater tendency for type 2 diabetes and higher cardiovascular risk have been identified in Japanese migrants who have acquired Brazilian habits [6,7].

The majority of studies focus on the effects of international migration, considering that risk factors for chronic diseases tend to be uniform within the same country yet differ between countries [8]. Vineis *et al*. [9], in a study of 1,400 individuals from the city of Turin and Varese province in Italy comparing natives of the North with migrants from the South, reported that the intake of saturated fatty acids and cholesterol was lower amongst migrants from the South, who also consumed more vegetables than individuals born in northern Italy. Also in Italy, different epigenetic signatures were observed between natives (North-Western) and migrants (South-to-North), possibly as an adaptive response to deal with environmental and lifestyle exposures discontinued from early life (perinatal exposures) with alterations resulting from migration [8].

The LIPGENE study showed that geographic location impacts dietary patterns, body mass index, plasma levels of fatty acids and urinary metabolic profile, with differences found among European regions (Northwest, Northeast and Southeast) [10]. In the EPIC (European Prospective Investigation into Cancer and Nutrition), different plasma concentrations of vitamin B-complex and other components of folate and homocysteine metabolism were observed amongst European regions [11].

Brazil, a continental country with disparate food availability and marked socioeconomic and environmental differences in five regions (North, Northeast, Mid-West, Southeast and South) has experienced significant migratory flows of Brazilians, particularly from the Northeast to the Southeast regions, and from the rural to the urban environment. The Northeast is a region subject to periods of drought and has one of the worst socioeconomic indices in the country, whereas the Southeast region has undergone a rapid process of industrialization and urbanization and has better social indices. Thus, the city of São Paulo, the largest metropolis in South America, was one of the cities which received the most migrants, in search of a better quality of life [12–15].

Studies with migrants are important because it is usually observed incorporation of the eating habits of the host population. However, unlike Japanese immigrants in Brazil who acquired Western dietary habits [6,7], the internal migrants, mainly those from the Northeast, due to their compromised socioeconomic status, restricted access to food, and low wages, they would not incorporate the food pattern of São Paulo. Therefore, we hypothesized that natives of São Paulo and Brazilian internal migrants have different dietary patterns.

In this context, in studies with dietary patterns approaches, the cumulative and interactive effects of diet can be take into account, representing more accurately the actual consumption besides having a broad applicability. Some controversial or non-significant results about the role of nutrition on health outcomes can be in part explained by the study of specific nutrients, foods or food groups rather than the study of dietary components combinations in a holistic way [16,17].

The objective of this study was to assess the differences and similarities in the dietary pattern between migrants (originally from Southeast and Northeast regions) and natives of São Paulo city, who took part in a population-based survey conducted in the city of São Paulo.

## Materials and methods

### Study population and sample selection

A population-based study with a cross-sectional design was carried out by applying a questionnaire at households and over the telephone to individuals living at permanent households located in the urban area of São Paulo (ISA-Capital). Two-stage cluster sampling was employed: census sectors and households, enabling estimation of a prevalence of 0.5, with error of 0.07, 95% confidence level and design effect of 1.5. Further details can be found elsewhere [18,19].

The first data collection was performed between 2008 and 2009. The information was obtained at households by trained interviewers who applied questionnaires (socioeconomic, lifestyle and dietary survey) to the randomly selected dwellers. The second data collection was conducted during 2009 and 2010 by performing another dietary survey, this time over the telephone, which entailed repeat application of the 24 hour (24-h) dietary recall.

The present study was approved by the Research Ethics Committee of the School of Public Health of the University of São Paulo (CAAE: 47629115.5.0000.5421) and all participants signed a free and informed consent form.

### Internal migration

**Fig 1:**
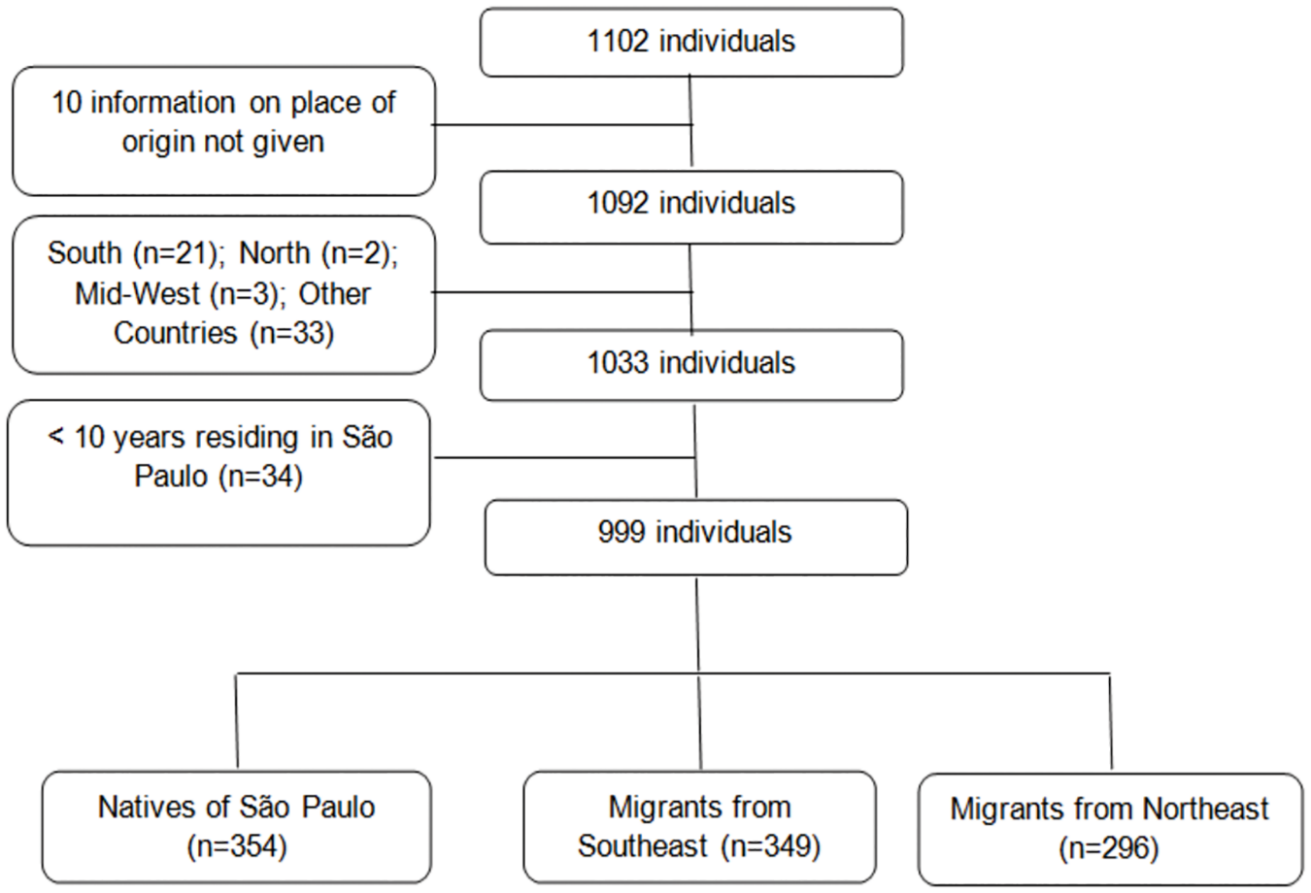
Description of the study sample selection process.

Using the socioeconomic questionnaires, information on the place of origin of the participants and length of time residing in the city was collected. Internal migrants were defined as individuals born outside São Paulo city (Southeastern capital city of São Paulo state) who had been living in the city for ten years or longer. Although Brazil comprises five geographic regions, the study focused on migrants from the Southeast [States of Rio de Janeiro, Minas Gerais, Espírito Santo and São Paulo (except for São Paulo city)] and the Northeast (States of Ceará, Rio Grande do Norte, Maranhão, Pernambuco, Bahia, Paraíba, Piauí, Alagoas and Sergipe), because they represent regions with different levels of industrialization, socioeconomic indicators and concentration of Gross National Product, factors distinguishing the regions since the wave of migration up to the present day. In addition, migrants from other regions and countries were excluded, given that the number of individuals from the two chosen regions was far greater than the remaining regions (South–n = 21, Mid-West–n = 3, North–n = 2 and other countries–n = 33). Of the initial 1102 individuals (≥ 20 years) that completed the dietary questionnaire, 999 remained in the final sample (Fig 1).

### Socioeconomic variables, reported morbidity and lifestyle

Demographic (age, sex, and self-declared race), socioeconomic (income *per capita* and head of household education level), lifestyle (physical activity, as measured by the international physical activity questionnaire–long version (IPAQ) [20], smoking and alcohol consumption), self-reported morbidity (hypertension and type 2 diabetes) and anthropometry (body weight (kg) and height (cm)) variables were collected and validated *a posteriori* [21–23]. The head of household was recognized by the study participant, and this information was used only to describe the study population.

Self-reported hypertension and diabetes were verified by asking the questions: "Do you have any chronic disease, any long-term illness or one that repeats itself with some frequency?"; "Hypertension (high blood pressure)?" or “Diabetes (if only gestational diabetes, select no)”. Those who reported having hypertension were also asked: "Who told you that you have high blood pressure?" or “Who told you that you have diabetes?” All individuals answered that a doctor made the diagnosis.

Anthropometric data were used to calculate Body Mass Index (BMI) and individuals were subsequently classified according to the BMI cut-off points recommended by the World Health Organization [24] for adults and the Pan American Health Organization (PAHO) for older adults [25].

### Dietary consumption

The dietary data were collected using two 24-h Dietary Recalls (one applied at the household and another over the telephone). The first 24-h recall was applied using the MPM (Multiple-Pass Method) [26], which seeks to keep the individual interested and engaged in the interview, helping them recall all items consumed and rendering the data more reliable [26]. The second 24-h recall was applied using the AMPM (Automated Multiple-Pass Method), which follows the same steps as the MPM, only the 24-h recall was applied by telephone coupled to the Nutrition Data System for Research software. The data collections were carried out on all days of the week spread throughout the four seasons of the year.

The data from the two 24-h recalls were keyed into the Nutrition Data System for Research software, which uses the data base from the food composition table of the United States Department of Agriculture—USDA [27]. The software has a wide range of functionalities and foods registered, which allow the analysis of nutrients, foods and eating events. The imputation of regional recipes in the software was made based on Brazilian tables of foods and preparations compositions [28,29]. Afterward, a detailed consistency analysis was conducted in order to identify and correct errors in the data imputed. The dietary energy density was estimated with the exclusion of foods consumed in the form of beverages (foods only method) [30].

### Dietary pattern

Around 1200 foods were reported in the nutritional questionnaires where these were grouped according to nutritional value, dietary habits of the São Paulo-born population and data from the literature, giving 34 food groups, similar to a previous study in the same population [18]. Foods or food groups consumed by less than 5% of the sample were excluded from the analyses. Usual dietary intake was estimated by the Multiple Source Method (MSM) in order to attenuate intrapersonal variance [31].

Factor and principal component analysis was employed to derive dietary patterns. Sample adequacy was checked using the Kaiser-Meyer-Olkin (KMO) index and Bartlett´s sphericity test, where values of KMO>0.50 and p<0.05, respectively, were considered acceptable. The number of factors (patterns) to be retained was determined as follows: initial criteria of *eigenvalues* > 1.0 and analysis of the Scree plot with subsequent interpretation of each factor. Data was then rotated using the Varimax method. The patterns were named according to the food groups which scored on each factor (factor loading ≥ 0.25 or ≤ -0.25). The criteria to label the dietary patterns in this study were based not only on previous publications using the same dataset who also derived dietary patterns [18, 32], but also on the scientific literature [33]. In previous studies developed in the ISA-Capital, the authors obtained similar dietary patterns with significant association with hypertension and other risk factors for cardiovascular diseases [18,32]. Subsequently, standardized factor score coefficients (mean of zero and a standard deviation of 1), i.e., the individual values of the factors, were estimated by regression approach. These scores indicated the degree to which each subject’s diet corresponded to the identified pattern. S1 Fig shows the spatial graphical representation of the derived factors.

### Statistical analysis

Socioeconomic, lifestyle, anthropometric variables, and self-reported diseases were expressed as means for continuous variables and as percentages for categorical variables, according to place of birth (natives of São Paulo, migrants from the Southeast and migrants from the Northeast). The Chi-squared and Student´s *t*-test or ANOVA test was used to compare groups.

The standardized scores obtained by the factor analysis were compared among groups using the ANOVA test and linear regression adjusted for confounding factors. The variables chosen for adjustments were sex, age, income *per capita*, self-declared race, smoking, body mass index, arterial hypertension, and energy intake. Values of standardized scores were expressed as mean and standard error or regression coefficient and 95% confidence interval.

All statistical analyses were conducted using the STATA package (Stata statistical software: release 13, StataCorp 2011, College Station, TX, USA), and a value of p < 0.05 was considered statistically significant.

## Results

Differences in income *per capita*, head of household education level, self-reported race, smoking habits, alcohol consumption, nutritional status, and prevalence of hypertension were found for place of birth of the participants in the ISA-Capital study. Length of time residing in São Paulo differed among migrants, proving longer in individuals from the Southeast (p<0.001). No differences for the variables sex, diabetes prevalence or practice of physical activity, and place of birth were found (Table 1).

**Table 1:** Socioeconomic and lifestyle characteristics among natives of São Paulo, migrants from the Southeast and migrants from the Northeast.

Socioeconomic variables	Natives of São Paulo n = 354	Migrants from Southeast n = 349	Migrants from Northeast n = 296	p*
Time residing in São Paulo, years**	-	44.9 (14.2)	33.6 (12.9)	**<0.001**
Age, years**	50.3 (18.3)^a^	59.3 (17.7)^b^	52.2 (18.6)^a^	**<0.001**
Sex, male	137 (38.7)	130 (37.2)	113 (38.2)	0.923
Income *per capita*, ≤ 1 MW	137 (38.7)	143 (41.0)	162 (54.7)	**<0.001**
Head of household education, ≤ 9 years	160 (45.8)	240 (69.6)	249 (85.3)	**<0.001**
Self-declared race, white	249 (70.5)	222 (63.6)	139 (47.0)	**<0.001**
**Lifestyle**				
Smoking habit, smokers	89 (25.1)	59 (16.9)	48 (16.2)	**0.011**
Alcohol consumption, yes	188 (53.1)	163 (46.7)	108 (36.5)	**<0.001**
Physical activity, Sufficiently Active	61 (17.3)	60 (17.2)	43 (14.6)	0.586
**Nutritional Status**				**0.003**
Underweight	30 (8.8)	49 (14.5)	25 (8.9)	
Normal	149 (43.6)	155 (45.7)	158 (56.4)	
Overweight	100 (29.2)	79 (23.3)	63 (22.5)	
Obese	63 (18.4)	56 (16.5)	34 (12.1)	
**Self-reported diseases**				
Hypertensive	100 (28.2)	148 (42.4)	121 (40.9)	**<0.001**
Diabetic	35 (9.9)	42 (12.0)	36 (12.2)	0.574

MW: Brazilian minimum wage (US$ 260.00).

*Chi-squared test (results are expressed in n(%));

**Student´s t-test or ANOVA test (results are expressed in mean (standard deviation)), with Tukey´s post-hoc. Different letters indicate significant differences.

Migrants from the Northeast had lower intakes of lipids, added sugar and fruit, vegetables and legumes compared with natives of São Paulo. Migrants from the Southeast had lower added sugar intake and energy density compared with natives of São Paulo (Table 2).

**Table 2:** Dietary intake among natives of São Paulo, migrants from the Southeast and migrants from the Northeast.

Dietary variables	Natives of São Paulo n = 354	Migrants from Southeast n = 349	Migrants from Northeast n = 296	p*
Energy, kcal	1689.1 (39.1)	1632.9 (37.5)	1599.0 (42.8)	0.274
Protein, g/1000kcal	43.3 (0.7)	44.7 (0.7)	45.5 (0.7)	0.089
Animal protein, g/1000kcal	28.2 (0.7)	29.1 (0.8)	29.8 (0.8)	0.339
Plant protein, g/1000kcal	15.1 (0.2)	15.6 (0.3)	15.6 (0.3)	0.259
Carbohydrate, g/1000kcal	126.2 (1.4)	125.3 (1.3)	128.1 (1.4)	0.362
Added sugar, g	45.5 (2.2)^b^	33.0 (1.6)^a^	36.7 (2.1)^a^	**<0.001**
Dietary fiber, g	16.5 (0.5)^a.b^	16.9 (0.5)^b^	15.4 (0.4)^a^	**0.048**
Lipid, g/1000kcal	35.2 (0.4)^b^	35.3 (0.5)^b^	33.1 (0.5)^a^	**0.002**
Saturated fat, g/1000kcal	10.7 (0.2)^a.b^	10.8 (0.2)^b^	9.9 (0.2)^a^	**0.019**
Monounsaturated fat, g/1000kcal	11.1 (0.2)^a.b^	11.2 (0.2)^b^	10.5 (0.2)^a^	**0.025**
Polyunsaturated fat, g/1000kcal	7.1 (0.1)	7.0 (0.1)	6.7 (0.1)	0.108
Cholesterol, mg	218.5 (8.9)^a.b^	223.6 (9.8)^b^	190.1 (7.9)^a^	**0.025**
Sodium, mg	3049.9 (79.9)	2933.7 (75.0)	2950.3 (84.3)	0.523
Number of meals	3.9 (0.1)	4.1 (0.1)	4.0 (0.1)	0.102
Glycemic index	55.4 (0.3)	54.8 (0.2)	55.0 (0.3)	0.226
Glycemic load, g	109.2 (2.8)	102 (2.5)	104.2 (2.9)	0.208
Fruit, vegetables and legumes, g	243.1 (14.3)^b^	255.7 (12.2)^b^	179.5 (11.3)^a^	**<0.001**
Energy density, kcal/g	1.8 (0.03)^b^	1.7 (0.03)^a^	1.9 (0.03)^b^	**0.001**

*ANOVA test, with Tukey´s post-hoc.; Different letters indicate significant differences. Values expressed as mean and standard error.

Three dietary patterns were derived: traditional, prudent and modern, which together explained 19.4% of the total variance in intake, with a KMO = 0.588 and Bartlett´s sphericity test < 0.001. The prudent pattern was characterized by consumption of salad dressings, vegetables, natural flavorings, fruits, whole-grain bread, white cheeses and juices. The traditional pattern had a positive factor loading for beans, rice, bread/toast/crackers, sugar, butter/margarine, coffee and whole milk and negative loading for whole-grain bread, reduced-fat/nonfat milk, and fruit. The modern pattern was characterized by consumption of sodas, pastries/sandwiches/pizzas, yellow cheeses, sauces, pastas, alcoholic beverages, sweets, processed meats, cold cuts, and canned vegetables (Table 3).

**Table 3:** Dietary pattern of migrants and natives residing in the city of São Paulo.

Dietary Patterns^#^		
Prudent	Traditional	Modern
Salad dressings (0.72)	Rice (0.64)	Sodas (0.52)
Vegetables (0.68)	Beans (0.62)	Pastries, sandwiches, pizzas (0.49)
Other vegetables (0.68)	Bread, toast, crackers (0.53)	Yellow cheeses (0.41)
Natural flavorings (0.42)	Sugar (0.50)	Sauces (0.41)
Fruits (0.38)	Butter, margarine (0.48)	Pastas (0.39)
Whole-grain bread (0.36)	Coffee, teas (0.32)	Alcoholic beverages (0.37)
White cheeses (0.33)	Whole milk (0.27)	Sweets (0.34)
Juices (0.32)	Fruits (-0.25)	Processed meats (0.32)
	Reduced-fat and nonfat milk (-0.29)	Cold cuts (0.32)
	Whole-grain bread (-0.30)	Canned vegetables (0.27)

Factor loadings ≥ 0.25 or ≤ -0.25 were considered significant; Factor loadings were expressed in parentheses.

^#^ Dietary pattern derived by factor analysis with extraction of factor loadings by principal components.

Overall, the factor loading of São Paulo native exhibited a stronger relationship of this population with the modern dietary pattern. For migrants from the Northeast, the average of factor loadings was positive for the traditional pattern, to a lesser degree, and negative for the prudent, and modern patterns. The greatest average factor loading of the migrants from the Southeast was positive for the prudent pattern (mean = 0.11). On adjusted regression analysis, compared to natives of São Paulo, migrants from the Southeast had an inversely proportional adherence to the modern pattern whereas migrants from the Northeast had an inverse association with the prudent and modern patterns and a positive association with the traditional pattern (Table 4).

**Table 4:** Dietary pattern among natives of São Paulo, migrants from the Southeast and migrants from the Northeast.

Dietary Pattern		Natives of São Paulo	Migrants from Southeast	Migrants from Northeast	p*
**Prudent**	Mean (SE)	0.09 (0.06)^b^	0.108 (0.05)^b^	-0.238 (0.05)^a^	**<0.001**
	β (95% CI)**	Ref.	-0.04 (-0.19;0.11)	**-0.29 (-0.45;-0.13)**	
**Traditional**	Mean (SE)	-0.104 (0.06)^a^	-0.028 (0.05)^a^	0.157 (0.05)^b^	**0.003**
	ß (95% CI)**	Ref.	0.13 (-0.01;0.26)	**0.22 (0.07;0.37)**	
**Modern**	Mean (SE)	0.249 (0.06)^b^	-0.068 (0.05)^a^	-0.217 (0.06)^a^	**<0.001**
	ß (95% CI)**	Ref.	**-0.18 (-0.31;-0.04)**	**-0.31 (-0.46;-0.17)**	

*ANOVA;

**Linear regression adjusted for age, sex, income *per capita*, self-declared race, smoking, body mass index, hypertension, and energy intake; Dependent variable: standardized scores. Different letters indicate significant differences; Values expressed as mean and standard error or regression coefficient (β) and 95% confidence interval (95% CI).

## Discussion

The objective of this study was to assess the differences and similarities in dietary intake of individuals (natives of Southeast and Northeast regions) who had migrated to São Paulo, the largest, most industrialized city in South America, and of natives of São Paulo city. The results found corroborate with the hypothesis that natives of São Paulo and Brazilian internal migrants have different dietary patterns and consumption, which can be associated with the differences in non-communicable disease rates mot only among natives and migrants, but also among different Brazilian regions and capitals. According to a recent publication, the prevalence of overweight and dyslipidemia were different between the Brazilian capitals. Type 2 diabetes and hypertension were less reported among capitals from North and Northeast, when compared with the city of Sao Paulo [34]. The same result was observed in the Brazilian National Health Survey in 2013: the North and Northeast regions presented lower prevalence of hypertension, diabetes, cardiovascular diseases and cancer compared to Southeast regions [35].

Migrants from the Northeast region were predominantly non-white, had ≤ 9 years´ education and income per capita of ≤ 1 minimum wage (MW). In this study, these migrants were characterized as the group with the worst socioeconomic level. This migrant group adhered to the traditional dietary pattern which consisted mainly of foods contained in the basic hamper of staples and are therefore more accessible, such as: rice, beans, biscuits/crackers, coffee and sugar. In addition, these migrants had lower consumption of fruit, vegetables, legumes and whole grains, which may be due to both the high cost of these items and also to their accessibility. Studies have shown that neighborhoods with low-income populations [36] and lower educational level [37] tend to have a lower density of supermarkets and fruit & vegetable markets coupled with a greater density of fast food outlets. Claro *et al*. [38] showed that in Brazil, the consumption of fruit, vegetables and legumes is inversely associated with income, and that a 1% increase in monthly income of Brazilian families was associated with a 0.04% increase in the proportion of fruit, vegetables and legumes in the total foods acquired. This same association has also been confirmed in developed countries [39, 40].

Data from the 2008/2009 Family Budget Survey reveals disparities in food consumption across Brazil´s major regions. Foods such as rice, beans, beef and whole milk, although consumed in all regions, are major contributors to dietary consumption in the Mid-west region, whereas consumption of potato and yoghurt was higher in the South and Southeast regions. Based on data from the National Health Survey [41,42] conducted in 2013, comparison of dietary consumption across the different regions of Brazil shows that the city of São Paulo has a higher consumption of sweet products and soft drinks than the Southeast and Northeast regions. The Northeast region on the other hand, has a lower prevalence of fruit, vegetable and legume consumption, corroborating the present findings which revealed adherence to the modern pattern (rich in sweet products and soft drinks) by natives of São Paulo and to the traditional pattern (negative factor loading for fruit) by migrants from the Northeast, thus evidencing a possible persistence in the dietary pattern of migrants´ region of origin.

In general, migrants move in order to secure a better life for themselves and their family. However, some studies have shown marked socioeconomic differences compared to the local population [43]. The majority of the study population migrated to São Paulo from the Southeast and Northeast regions between 1960 and 1980, and are therefore representative of the migratory process in Brazil. Compared to the native São Paulo population, significant differences in income, education and race are evident, proxy variables of unfavorable socioeconomic status.

Evidence shows that, hand in hand with the development of the food industry [44], higher income facilitates access to new foods which can lead to overconsumption of foods and beverages rich in sugar, salt and fats [45–47], and that internal migration from rural to urban areas is associated with greater intake of energy, macronutrients [48,49] and high-sugar beverages and fried snacks [50].

The health effects of migration from rural to urban areas has been described by other countries (i.e India and Peru). In India, those women who migrated from rural to urban areas were more likely to be overweight/obese (OR: 1.50, CI 95%: 1.36–1.65), in comparison with rural non-migrants [51]. In addition, migration was associated with increased dietary intake of fat and with decreased physical activity levels and, consequently, higher prevalence of obesity and diabetes were found among migrants when compared to rural non-migrants [52]. Also, the time of exposition to the urban environment was associated with an increase in cardiometabolic risk factors [53]. In a longitudinal study in Peru, residents of urban areas (locals and migrants from rural areas) had higher incidence of obesity when compared with rural non-migrants [54]. However, despite the rural migrants having similar risk factors for cardiovascular diseases when compared to the urban population [55], in 5 years they have similar mortality from cardiovascular diseases with the rural population who never migrated [56].

Natives of São Paulo can be characterized as a group with a high family income and greater prevalence of overweight and obesity. This group also contains a higher number of individuals who are smokers and consumers of alcoholic beverages. These individuals exhibit greater adherence to the modern dietary pattern, marked by the presence of convenience foods: pasta, canned vegetables, processed meats, yellow cheeses, sweet products, soft drinks, alcoholic beverages, cold cuts, and sauces. The purchase of processed meat products, ready-to-eat meals, soft drinks, and alcoholic beverages is also directly linked with income, where individuals with higher spending power consume these items more frequently [57].

The migrants from the Southeast were older overall, which may explain the negative association with the modern pattern and the positive factor loading with the prudent dietary pattern, and also had lower intake of added sugar, higher consumption of fruit, vegetables and legumes and lower energy density. This population had a higher prevalence of hypertension, a factor also associated with age. Although the cross-sectional design of the study precluded investigation of causal relationships, it may be the case that once diagnosed with the condition, these individuals may be pay more attention to their health and hence to their diet [18].

The healthy migrant hypothesis can potentially explain the healthier behavior of migrants from the Southeast [5]. This group of people probably already had better socio-demographic conditions and a healthier dietary pattern when they decided to carry out a move, when compared with those who remained in the home region. The same can be seen among South Asian migrants living in Canada. These migrants reported increased dietary intake of fruits and vegetables and decreased dietary intake of fried foods [58].

The main limitation of the study was the absence of information on the age at which individuals migrated, since this variable may have an important effect on the risk of diseases mediated by exposures in early life or by genetic factors [5]. However, information on length of stay in the city of São Paulo was collected, with the exclusion of individuals who had lived in the city for less than 10 years. Thus, by comparing average time of residence and mean age of the migrants, it can be deduced that most migrants left their places of birth during childhood/adolescence. The definitions of migration and period of residence available in the literature are in need of refinement. In the present study, differences in dietary patterns were found between migrants who had been living in the city for 10 years and natives of São Paulo. Previous studies employing the same cut-off point have reported differences in risk for obesity and type 2 diabetes, an effect which differed between sexes [52], although no major statistical difference in dietary intake was noted between decades [48].

Based on available data, the effect of migration on dietary intake cannot be verified owing to an absence of information on food intake practices in the city of origin. Further studies comparing diets in migrants´ home towns against that of the current place of residence are needed, allowing changes in eating habits and culture after migration to be identified.

To the best of our knowledge, this is the first study to observe differences in dietary patterns between internal migrants and natives using factor and principal component analysis in a population-based sample in Brazil. In contrast, this method has been criticized due to its subjective nature of decisions about food grouping, number of factors extracted, rotation methods, and labelling [16, 17]. Consequently, it is challenging to replicate the results and stablish a comparison between studies and populations [59]. However, the same dietary patterns were found in the present study as in a previous publication using the ISA-Capital data, even after exclusion of individuals, thereby evidencing the robustness of the analysis [18].

In summary, the present results confirmed that internal migrants and natives living in South America´s largest metropolis have different dietary patterns. This finding underscores the need to investigate the relationship between migration and the health/disease process and its relationship with diet. The use of objective markers of diet such as biomarkers and metabolites, as well as data on genetic ancestry, can further advance the study of migration and its implications. These results highlight the need for policies of social inclusion aimed at migrants. In this study, migration was associated with socioeconomic and dietary differences, despite the fact that migration had taken place many years earlier.

## Supporting information

S1 Fig Spatial representation of the relationships between derived factors and the food groups of the study of the dietary patterns. (TIF)
